# Severe Lymphopenia as a Predictor of COVID-19 Mortality in Immunosuppressed Patients

**DOI:** 10.3390/jcm10163595

**Published:** 2021-08-15

**Authors:** María Martínez-Urbistondo, Ángela Gutiérrez-Rojas, Ane Andrés, Isabel Gutiérrez, Gabriela Escudero, Sonia García, Andrea Gutiérrez, Enrique Sánchez, Jesús Herráiz, Sara De La Fuente, Alejandro Callejas, Carmen De Mendoza, Víctor Moreno-Torres

**Affiliations:** Internal Medicine Department, Hospital Universitario Puerta de Hierro Majadahonda, 28222 Madrid, Spain; angelagutierrezrojas@gmail.com (Á.G.-R.); aneandres60@gmail.com (A.A.); isabel_gut_mar@hotmail.es (I.G.); gabriela.escuderolopez@gmail.com (G.E.); soniagpr0@gmail.com (S.G.); a.gutierrezv@hotmail.com (A.G.); sanchezchica@gmail.com (E.S.); jesusherraizjimenez@gmail.com (J.H.); sfuentem@salud.madrid.org (S.D.L.F.); alejandro.calleja@salud.madrid.org (A.C.); cmendoza.cdm@gmail.com (C.D.M.); victor.moreno.torres.1988@gmail.com (V.M.-T.)

**Keywords:** COVID-19, immunosuppression, severe lymphopenia

## Abstract

Background. Coronavirus disease 2019 (COVID-19) has a high mortality in certain group of patients. We analysed the impact of baseline immunosuppression in COVID-19 mortality and the role of severe lymphopenia in immunocompromised subjects. Methods. We analysed all patients admitted with COVID-19 in a tertiary hospital in Madrid between March 1st and April 30th 2020. Epidemiological and clinical data, including severe lymphopenia (<500 lymphocytes/mm^3^) during admission, were analysed and compared based on their baseline immunosuppression condition. Results. A total of 1594 patients with COVID-19 pneumonia were hospitalised during the study period. 166 (10.4%) were immunosuppressed. Immunocompromised patients were younger (64 vs. 67 years, *p* = 0.02) but presented higher rates of hypertension, diabetes, heart, neurological, lung, kidney and liver disease (*p* < 0.05). They showed more severe lymphopenia (53% vs 24.1%, *p* < 0.001), lower SapO_2_/FiO_2_ ratios (251 vs 276, *p* = 0.02) during admission and higher mortality rates (27.1% vs 13.5%, *p* < 0.001). After adjustment, immunosuppression remained as an independent factor related to mortality (Odds Ratio (OR): 2.24, *p* < 0.001). In the immunosuppressed group, age (OR = 1.06, *p* = 0.01), acute respiratory distress syndrome (ARDS) (OR = 12.27, *p* = 0.017) and severe lymphopenia (OR = 3.48, *p* = 0.04) were the factors related to high mortality rate. Conclusion. Immunosuppression is an independent mortality risk factor in COVID-19. Severe lymphopenia should be promptly identified in these patients.

## 1. Introduction

Coronavirus disease 2019 (COVID-19) emerged in China in December 2019 and rapidly spread worldwide, being declared as a pandemic by the World Health Organization (WHO) in March 2020 [1,2]. By mid-year 2021, more than 176 million confirmed cases with over 3.8 million deaths have been attributed to this pathogen. Two different phases have been described in SARS-CoV-2 infection [3]. The initial viral period lasts for 8 to 10 days and has a self-limited course in almost 80–90% of cases. The second one, defined as “the inflammatory stage”, correlates with the host immune response and a massive inflammation mediators release known as the cytokine storm. As a consequence, endothelial cells, macrophages, monocytes and lymphocytes are involved with a wide spectrum of clinical manifestations [4].

However, while this hyperinflammation and cytokine storm seem to lead to the acute respiratory distress syndrome (ARDS) and severe COVID-19 disease, the role of baseline immunosuppression in COVID-19 pandemic has not been defined yet despite recent studies that have assessed this matter [5,6,7,8]. On one hand, Giannakoulis et al. [6], reported in a wide meta-analysis that patients with cancer presented more Intensive Care Unit (ICU) admission and death. On the other hand, Minotti et al. [7] identified better outcomes in this population, proposing a possible protective role of a potential weaker immune response. In addition, immunosuppression probably entails a different natural course of the disease, atypical presentations and the higher probability of other complications such as bacterial superinfections [9], leading to a more complex management of this population. Indeed, deep lymphopenia is observed in most immunosuppressed patients as a result of a defective immune response. Multiple models revealed lymphocyte count as a useful prognostic biomarker in SARS-CoV-2 infection [10,11]. In this context, underlying immunosuppression may have synergistic effect in SARS-CoV-2 mortality rates [12].

Therefore, the aim of this study was to evaluate the prognostic role of immunosuppression in COVID-19 patients. In addition, we analysed the mortality risk factors in our immunosuppressed population as well as the influence of severe lymphopenia in this specific cohort.

## 2. Materials and Methods

### 2.1. Study Design and Patients

This retrospective observational cohort study was performed at Hospital Puerta de Hierro-Majadahonda, a large tertiary university hospital located in Madrid. The study population consisted of 1594 adult patients who were admitted between 1st March and 30th April because of interstitial pneumonia detected by chest X-ray due to suspected or confirmed SARS-CoV-2. Both real time polymerase chain reaction (RT- PCR) on nasopharyngeal exudate confirmed SARS-CoV-2 infection and suspected SARS-CoV-2 interstitial pneumonia (in the absence of other causes) were included according to the WHO criteria [13]. Follow-up continued to June 30th, 2020. The study was approved by the hospital’s Research Ethics Commission and all patients were requested their consent to register their clinical information into a database for epidemiological studies. The subjects were then distributed and compared according to immunosuppression condition considering non-immunosuppressed as controls. Mortality was the main outcome as the chief aim of the investigation is the identification of prognostic determinants in COVID-19 evolution.

### 2.2. Data Collection

Electronic medical records for all hospital admitted patients with COVID-19 pneumonia were reviewed. Main demographics, baseline comorbidities, including immunosuppression and immunosuppressive treatment, minimum lymphocyte counts during hospitalisation, immunosuppressive treatment received to treat COVID-19, ARDS and outcomes, were collected directly from the electronic medical record. All data were registered by a primary reviewer and subsequently checked by at least two senior physicians.

### 2.3. Definitions

Immunosuppression was defined either as the presence of hematologic disease (active lymphoproliferative, myeloproliferative disorders or bone marrow transplantation), solid organ transplantation, active and disseminated solid organ neoplasm or any condition, including autoimmune disease (e.g., Systemic Lupus Erythematosus, Sjögren Syndrome, inflammatory bowel disease) that had required immunosuppressive treatment for at least 3 months before admission due to COVID-19. Immunosuppressive treatment was considered when the patient was either receiving active treatment at the moment of admission, including equivalent prednisone doses above 5 mg, or had received chemotherapy or immunotherapy 6 months before disease onset.

Regarding clinical data, severe lymphopenia in the context of COVID-19 was defined as minimum lymphocyte count below 500/mm^3^ during admission [14,15]. On the other hand, ARDS and its severity were defined according to the Berlin definition [16]. In the patients whose partial pressure of oxygen (PaO_2_) was unavailable, SapO_2_/FiO_2_ ratio was used to assess ARDS and severity [17]. Mild ARDS was considered when PaO_2_/FiO_2_ ratio was >200 mmHg or SapO_2_/FiO_2_ >235 mmHg, moderate when PaO_2_/FiO_2_ ratio was >100 mmHg or SapO_2_/FiO_2_ > 160 mmHg and severe when PaO_2_/FiO_2_ ratio was ≤100 mmHg.

### 2.4. Statistical Analysis

Descriptive analyses were performed through the mean (standard deviation, SD) for quantitative variables and absolute (and relative) frequencies for the categorical. A univariate analysis was performed comparing those characteristics for patients who were immunosuppressed versus those who did not, and also, between (immunosuppressed) survivors and non-survivors by means of chi-square test in case of categorical variables and Mann-Whitney’s U or Student t-test for numerical variables depending on their distributions and performing the Levène test. Potential confounding variables were entered into different multivariable logistic regression analyses to identify factors related to mortality. For all analyses, significance level was defined as a *p*-value below 0.05. Statistical analysis was performed using SPSS software version 26.0 (IBM, Madrid, Spain).

## 3. Results

### 3.1. Patients’ Characteristics

A total of 1594 patients admitted because of suspected or confirmed SARS-CoV-2 pneumonia between March and April 2020 were analysed. Mean age was 65 years old, 62.1% were male and 87.2% had a positive RT-PCR for SARS-CoV-2 at the time of admission. Overall, 166 (10.4%) were immunosuppressed (Table 1). Autoimmune disease was the most frequent cause of immunosuppression (51 patients, 39.2%), followed by hematologic disease (35 patients, 21.1%), solid organ neoplasm (32 patients, 19.3%) and solid organ transplantation (30 patients, 18.1%). Overall, 40.4 % of immunosuppressed patients were receiving steroid therapy, 18.1 % biological agents, 15.1% calcineurin inhibitors, 9.6% mycophenolate and active chemotherapy prior to admission.

Average age was lower in the immunosuppressed group in comparison to not immunocompromised patients (64 vs. 67 years, *p* = 0.02). However, they presented higher rates of baseline comorbidities such as high blood pressure (52.4% vs. 42.9%, *p* = 0.02), diabetes mellitus (27.1% vs. 16.5%, *p* = 0.001), heart disease (24.7% vs. 16.0%, *p* = 0.01), neurological disease (20.5% vs. 13.4%, *p* = 0.02), lung disease (21.7% vs. 14.8%, *p*= 0.03), kidney disease (20.5% vs. 5.5%, *p* < 0.001) and liver disease (10.8% vs. 2.1%, *p* < 0.001) (Table 1).

Concerning disease severity, no differences were observed in ARDS rates during admission (78.3% vs. 75.8%, *p* = 0.50) or minimum mean lymphocyte count (1227/mm^3^ vs. 1060/mm^3^, *p* = 0.551); but patients with prior immunosuppression presented lower SapO_2_/FiO_2_ ratios (251 vs. 276, *p* = 0.02) and more severe lymphopenia (53%, vs. 24.1%, *p* < 0.001). They received steroid therapy more frequently during admission (70.5% vs. 56.7%, *p* = 0.001). Immunocompromised subjects presented infectious complications more frequently (*p* < 0.001) (Table 2).

Considering outcomes, immunosuppressed patients had a longer hospital average stay (13 vs. 9 days, *p* < 0.001) and higher readmission rates after discharge (10.8% vs. 5.1%, *p* = 0.007). Overall mortality was 15.1%, being significantly higher in patients with baseline immunosuppression (28.9 % vs. 13.5%, *p* < 0.001) (Table 2).

### 3.2. Immunosuppression as an Independent Factor Related to Mortality

Since a higher proportion of individuals with immunosuppression died during admission, a multivariate analysis to identify the mortality risk factors was performed (Table 3). Mortality was associated with previous immunosuppression (OR: 2.24, 95%CI= 1.37–3.65) as well as other known baseline comorbidities, including age (OR:1.11, 95%CI:1.09–1.13), neurological disease (OR: 2.79, 95%CI: 1.89–4.11), kidney disease (OR: 2.62, 95%CI: 1.58–4.34) and by the presence of ARDS (OR: 14.06, 95% CI 4.80–41.20) and severe lymphopenia (OR: 2.95, 95% CI 2.03–4.30).

### 3.3. Prognostic Risk Factors of Mortality in Immunosuppressed Patients

In order to identify the prognostic risk factors in immunosuppressed patients (166 individuals), as well as the impact of lymphopenia, univariate and multivariate analyses were performed in this population. Immunosuppressed non-survivors were significantly older than survivors (73.9 vs 64.6 years, *p* < 0.001). In addition, in the univariate analysis (Table 4), mortality in immunosuppressed patients was associated with heart disease (*p* = 0.003), kidney disease (*p* = 0.04), severe lymphopenia (*p* < 0.001) as well as the presence of ARDS (*p* < 0.001) and SapO_2_/FiO_2_ ratio (*p* < 0.001).

In the multivariate analysis (Table 5), severe lymphopenia associated a three-fold increased mortality risk in the immunosuppressed subjects (OR = 3.48, 95% CI 1.49–8.12), along with age (OR = 1.06, 95% CI 1.02–1.09) and ARDS (OR = 12.27, 95% CI 1.56–96.41).

## 4. Discussion

Immunosuppression has a controversial role in COVID-19 pneumonia, because of the key hyperinflammation cascade determining ARDS appearance and the proper defective host immune response in these particular subjects [18]. In this study, we assessed the impact of immunosuppression in COVID-19 mortality and the added value of lymphopenia in immunosuppressed individuals as a prognostic marker in one of the most affected regions by COVID-19 during the first wave [9].

In our cohort, immunosuppressed patients presented higher comorbidities and more severe disease. However, immunosuppression remained as an independent condition for fatal outcomes after adjustment by the factors previously described [19]. Emerging studies also show a worse trend among COVID-19 patients with immunosuppressant conditions [20]. This fact might suggest that immunosuppression intrinsically involves poor prognosis as published by Williamson et al. in a wide transplanted cohort [21] and Giannakoulis et al. in a meta-analysis based in patients diagnosed with cancer [6]. Consequently, ARDS severity and worse outcomes could be related with the dysfunctional immune response, rather than with direct viral damage to the lungs. Similarly, Ritchie et al. considered this disorder as a “double-edged sword” given a delayed SARS-CoV-2 clearance and a subsequent longer disease course [22]. In addition, a systematic review proposed these subjects as possible viral reservoirs in this context given the previously described data [7]. A pro-inflammatory previous status and a deficient counter-regulation to the cytokine delivery might aggravate clinical manifestations. In fact, persistent COVID-19 has been reported in patients receiving immunosuppressive therapies for several causes as cancer, solid organ transplantation or autoimmune diseases [23,24,25].

Therefore, immunosuppression may be an underlying condition explaining some deaths in younger and more comorbid subjects. However, not every immunosuppressed individual reacts equally to SARS-CoV-2 infection. Almost 80% of fatal cases in our immunocompromised population presented severe lymphopenia, even though there were not statistically significant differences in total lymphocyte counts. Decreased lymphocyte count has been published as a viral infection biomarker [26,27,28]. In previous literature, 63–85% SARS-CoV-2 infected patients showed lymphopenia, up to 20% deeper in those who died [29]. This fact could be partially explained by impaired myelopoiesis and lymphohematopoiesis in COVID-19 necropsies. Concomitantly, lymphocyte redistribution has been recognised as a physiological response in viral pathologies lowering peripheral levels [30]. In immunocompromised individuals, a prolonged virus exposure might involve T-cell and natural killer cells exhaustion with an even more marked lymphopenia and an uncontrolled hypercytokinemia [31,32]. Therefore, severe lymphopenia may reflect a basal defective immune response to an aggressive viral infection, which unfortunately results in increased mortality rates in this specific population. Considering the diversity of our immunosuppressed cohort, we could define lymphopenia as a global mortality risk factor in these patients, independently of its trigger. Pharmacological targets or underlaying disease activity have not been tested in a more detailed analysis, but in the light of the conclusions this finding could possibly be a class effect derived of immunosuppression itself. Nevertheless, more investigations are required in this field. Lymphocyte count is an accessible and non-invasive determination that could probably be helpful to implement early and targeted therapeutic measures, in this concrete scenario. In addition, these findings confirm the requirement of specific management protocols for immunosuppressed patients with COVID-19, to avoid the excessive immunosuppression and lymphopenia that entail a fatal outcome.

Possible limitations include that the study is retrospective, cross-sectional and single-center and the sample (166 patients) may not be representative of the whole immunosuppressed population. In addition, it should be considered that immunosuppressed patients were analysed altogether in a heterogeneous population. As a consequence, the intensity and type of immunosuppression was not properly assessed. However, the described results are plausible with the previous published literature. Low statistical power could be responsible for some of the non-significant outcomes. We tried to minimise bias including the potential confounding variables in multivariate models. As a strong point, lymphocyte count has demonstrated to be a reliable marker in SARS-CoV-2 infection in other analyses, but this investigation contributes to identify a new role for this biomarker that has not been described before. Furthermore, sensitivity analysis allowed to discriminate the involvement of specific variables in the outcomes.

In summary, immunosuppression and severe lymphopenia in immunosuppressed patients might have a key role as predictors of fatal events in the COVID-19 pandemic. These approaches implementation will elicit promising outcomes in personalised medicine. In view of these results, the detection of severe lymphopenia in immunocompromised individuals should probably be considered as a red flag in usual clinical practice. Thus, the inclusion of these criteria in hospital protocols for an early intensive therapeutic approach could be a useful measure for reducing mortality in this vulnerable patient profile. As a concluding remark, this research will help to provide guidelines where the immunosuppression condition should be taken into account.

## Figures and Tables

**Table 1:** Baseline conditions of the study population *.

	GLOBAL	NON-IS (*n* = 1428)	IS (*n* = 166)	*p*-Value
**Baseline Conditions**				
**Age (*N*, %)**	65 (15)	67 (14)	64 (15)	**0.02**
Male sex (*N*, %)	990 (62.1)	887 (62.1)	103 (62.0)	1
**HBP (*N*, %)**	699 (43.9)	612 (42.9)	87 (52.4)	**0.02**
**DM (*N*, %)**	281 (17.6)	236 (16.5)	45 (27.1)	**0.001**
**Obesity (*N*, %)**	424 (35.2)	386 (36.1)	38 (27.9)	**0.001**
**Heart disease (*N*, %)**	270 (16.9)	229 (16.0)	41 (24.7)	**0.01**
**Neurological disease (*N*, %)**	225 (14.1)	191 (13.4)	34 (20.5)	**0.02**
**Lung disease (*N*, %)**	248 (15.6)	212 (14.8)	36 (21.7)	**0.03**
**Kidney disease (*N*, %)**	112 (7.0)	78 (5.5)	34 (20.5)	**<0.001**
**Liver disease (*N*, %)**	48 (3)	30 (2.1)	18 (10.8)	**<0.001**

* IS: Immunosuppressed. HBP: High Blood Pressure. DM: Diabetes Mellitus. Qualitative variables are expressed in number and proportion. Quantitative variables are expressed in mean and standard deviation. Statistically significant results are remarked in bold.

**Table 2:** Clinical characteristics, treatment received and outcomes during admission *.

	GLOBAL	NON-IS (*n* = 1428)	IS (*n* = 166)	*p*-Value
**Clinical Characteristics**				
Minimum lymphocyte count	1078 (3393)	1060 (3244)	1227 (4476)	0.551
(cells/mm^3^), (Mean, SD)				
**Severe Lymphopenia (*N*, %)**	432 (27.2)	344 (24.1)	88 (53)	**<0.001**
**Sap02/FiO2 ratio (Mean, SD)**	273 (124)	276 (123)	251 (131)	**0.02**
**ARDS (*N*, %)**	1212 (76.1)	1082 (75.8)	130 (78.3)	0.5
Mild (*N*, %)	660 (41.4)	604 (42.3)	56 (33.7)	**0.04**
Moderate (*N*, %)	420 (26.3)	365 (25.6)	55 (33.1)	**0.04**
Severe (*N*, %)	129 (8.1)	110 (7.7)	19 (11.4)	0.1
**Infectious complications**	135 (8.5)	96 (6.7)	39 (23.5)	**<0.001**
**Treatment Received during Admission**				
**Steroids (*N*, %)**	926 (58.1)	809 (56.7)	117 (70.5)	**0.001**
Tocilizumab (*N*, %)	300 (18.8)	260 (18.2)	40 (24.1)	0.07
**Anakinra (*N*, %)**	42 (2.6)	30 (2.1)	12 (7.2)	**0.001**
**Outcomes**				
ICU admission (*N*, %)	110 (6.9)	93 (6.5)	17 (10.2)	0.07
**In-hospital stay (Mean, SD)**	9.3 (10.1)	9 (9)	13 (14.0)	**<0.001**
**Readmission (*N*, %)**	91 (5.7)	736 (5.1)	18 (10.8)	**0.007**
**Death (*N*, %)**	241 (15.1)	193 (13.5)	45 (27.1)	**<0.001**

* IS: Immunosuppressed. ARDS: Acute Respiratory Distress Syndrome. ICU: Intensive Care Unit. Qualitative variables are expressed in number and proportion. Quantitative variables are expressed in mean and standard deviation. Statistically significant results are remarked in bold.

**Table 3:** Multivariate analysis for factors associated with mortality in 1594 COVID-19 hospitalised patients *.

	Odds Ratio	95% Confidence Interval
**Age**	**1.11**	**1.09–1.13**
HBP	1.1	0.74–1.66
DM	1.33	0.90–1.96
Heart disease	1.26	0.85–1.887
**Neurological disease**	**2.79**	**1.89–4.11**
Lung disease	0.75	0.47–1.18
**Kidney disease**	**2.62**	**1.58–4.34**
Liver disease	1.72	0.73–4.03
**Immunosuppression**	**2.24**	**1.37–3.65**
**Severe lymphopenia**	**2.95**	**2.03–4.30**
**ARDS**	**14.06**	**4.80–41.20**
Infectious complications	1.1	0.65–1.87
Steroids	0.68	0.45–1.03

* HBP: High Blood Pressure. DM: Diabetes Mellitus. SON: Solid organ neoplasm. SOT: Solid organ transplantation. ARDS: Acute Respiratory Distress Syndrome. Statistically significant results are remarked in bold.

**Table 4:** Baseline conditions, clinical characteristics and outcomes of immunosuppressed patients *.

	Survivors (*n* = 118)	Non-Survivors (*n* = 48)	*p*-Value
**Baseline Conditions**			
**Age (*N*, %)**	64.6 (12.4)	73.9 (14.5)	**<0.001**
Male sex (*N*, %)	73 (61.9%)	30 (62.5%)	1
HBP (*N*, %)	56 (47.5%)	31 (64.6%)	0.059
DM (*N*, %)	27 (22.9%)	18 (37.5%)	0.082
Obesity (*N*, %)	26 (26.5%)	12 (31.6%)	0.671
**Heart disease (*N*, %)**	21 (17.8%)	20 (41.7%)	**0.003**
Neurological disease (*N*, %)	24 (20.3%)	10 (20.8%)	1
Lung disease (*N*, %)	27 (22.9%)	9 (18.8%)	0.679
**Kidney disease (*N*, %)**	19 (16.1%)	15 (31.3%)	**0.035**
Liver disease (*N*, %)	12 (10.2%)	6 (12.5%)	0.783
Immunosuppression causes			
Autoimmune disease	51 (43.2%)	14 (29.2%)	0.115
Hematologic	22 (18.6%)	13 (27.1%)	0.294
Solid organ neoplasm	19 (16.1%)	13 (27.1%)	0.129
Solid organ transplantation	22 (18.6%)	8 (16.7%)	0.828
Others	4 (3.4%)	0 (0%)	0.325
**Severe lymphopenia**	50 (42.4%)	38 (79.2%)	**<0.001**
**Sap02/FiO2 ratio (Mean, SD)**	298 (119)	134 (77)	**<0.001**
**ARDS (*N*, %)**	83 (70.3%)	47 (97.9%)	**<0.001**
**Mild (*N*, %)**	52 (44.1%)	4 (8.3%)	**<0.001**
**Moderate (*N*, %)**	24 (20.3%)	31 (64.6%)	**<0.001**
**Severe (*N*, %)**	7 (5.9%)	12 (25%)	**<0.001**

* HBP: High Blood Pressure. DM: Diabetes Mellitus. ARDS: Acute Respiratory Distress Syndrome. Qualitative variables are expressed in number and proportion. Quantitative variables are expressed in mean and standard deviation. Statistically significant results are remarked in bold.

**Table 5:** Multivariate analysis for factors associated with mortality in COVID-19 immunosuppressed hospitalised patients *.

	OR	95% CI
**Age**	**1.06**	**1.02–1.09**
Heart disease	1.96	0.82–4.73
Kidney disease	1.47	0.58–3.71
**Severe lymphopenia**	**3.48**	**1.49–8.12**
**ARDS**	12.27	**1.56–96.41**

* OR: Odds Ratio. CI: Confidence interval. ARDS: Acute Respiratory Distress Syndrome. Statistically significant results are remarked in bold.

## Data Availability

The data are filed in the SELENE platform **(**SELENE System, Cerner Iberia, S.L.U, Madrid, España).
